# Neurotoxic Anatoxin-a Can Also Exert Immunotoxicity by the Induction of Apoptosis on *Carassius auratus* Lymphocytes *in vitro* When Exposed to Environmentally Relevant Concentrations

**DOI:** 10.3389/fphys.2020.00316

**Published:** 2020-04-15

**Authors:** Yuchi Zhong, Lilai Shen, Xueping Ye, Dongren Zhou, Yunyi He, Yan Li, Ying Ding, Weiqin Zhu, Jiafeng Ding, Hangjun Zhang

**Affiliations:** ^1^School of Life and Environmental Sciences, Hangzhou Normal University, Hangzhou, China; ^2^Zhejiang Institute of Freshwater Fisheries, Huzhou, China

**Keywords:** apoptosis, ANTX-a, oxidative stress, antioxidant enzymes, fish lymphocytes

## Abstract

Hazardous anatoxin-a (ANTX-a) is produced by freshwater algal blooms worldwide, which greatly increases the risk of consumer exposure. Although ANTX-a shows widespread neurotoxicity in aquatic animals, little is known about its mechanism of action and biotransformation in biological systems, especially in immunobiological models. In this study, transmission electron microscopy results showed that ANTX-a can destroy lymphocytes of *Carassius auratus in vitro* by inducing cytoplasmic concentration, vacuolation, and swollen mitochondria. DNA fragmentations clearly showed a ladder pattern in agarose gel electrophoresis, which demonstrated that the apoptosis of fish lymphocytes was caused by exposure to ANTX-a. Flow cytometry results showed that the apoptotic percentage of fish lymphocytes exposed to 0.01, 0.1, 1, and 10 mg/L of ANTX-a for 12 h reached 18.89, 22.89, 39.23, and 35.58%, respectively. ANTX-a exposure induced a significant increase in reactive oxygen species (ROS) and malonaldehyde (MDA) in lymphocytes. The activities of superoxide dismutase (SOD), catalase (CAT), glutathione reductase (GR), glutathione peroxidase (GPx), and the glutathione (GSH) content of the 0.01 mg/L ANTX-a-treated group decreased significantly by about 41, 46, 67, and 54% compared with that of the control group (*p* < 0.01), respectively. Although these observations were dose-dependent, these results suggested that ANTX-a can induce lymphocyte apoptosis via intracellular oxidative stress and destroy the antioxidant system after a short exposure time of only 12 h. Besides neurotoxicity, ANTX-a may also be toxic to the immune system of fish, even when the fish are exposed to environmentally relevant concentrations, which clearly demonstrated that the potential health risks induced by ANTX-a in aquatic organisms requires attention.

## Introduction

Cyanotoxins are toxic secondary metabolites produced by algal blooms, which are widely distributed in fresh water in various countries and regions of the world (Du et al., 2019; Ruibal-Conti et al., 2019). Several studies have shown that cyanotoxins, such as anatoxin-a (ANTX-a), and microcystins can bioaccumulate in aquatic biota, which poses a huge threat to the biological health and ecological environment, especially that of aquatic organisms (Ibelings and Havens, 2008). Svircev et al. (2019) investigated that there were 183 cases of cyanotoxin poisoning in humans and animals as of 2019. ANTX-a, chemically identified as 2-acetyl-9-azabicycle[4.2.1]non-2-ene (Smith and Lewis, 1987), is a neurotoxin that can be isolated from different genera of cyanobacteria, such as *Oscillatoria*, *Anabaena flos-aquae*, *Aphainizomenon*, *Cylindrospermum*, etc. (Cires and Ballot, 2016). Although microcystins seem to be more common than ANTX-a, the latter cause more organism poisonings in Europe, Australia, and North America than those caused by microcystins (Fitzgeorge et al., 1994). Dog and cattle poisonings related to ANTX-a were reported in six places in Canada between 1961 and 1975 (Trainer and Hardy, 2015). ANTX-a is becoming increasingly recognized as a potential risk to both animal and human health, which has been incorporated into laws and regulations regarding the water security of several countries (Rodriguez et al., 2017). In 2015, the U.S. government passed an amendment to the “Drinking Water Protection Act” (PL 114-45) that requires the implementation of the best available technology to assess the risks to human health of public water polluted by cyanotoxins such as ANTX-a (Metcalf et al., 2018).

ANTX-a is abundant in the environment and leads to detrimental effects on various organisms through drinking water and food. For instance, Carneiro et al. (2015) revealed that ANTX-a can damage proteins playing toles in carbohydrate metabolism, cell structure maintenance, stress response, etc., of male and female zebrafish. As hazardous neurocyanotoxins, ANTXs are strong agonists of the neural nicotinic cholinergic receptor existing in the postsynaptic membrane (Loftin et al., 2016). Animals reportedly die from exposure to ANTX-a following toxic response to acetylcholine receptor inhibition at neuromuscular junction (Carneiro et al., 2015). Chia et al. (2019) revealed that high-dose injection or ingestion of ANTX-a can cause fatal suffocation in mammals and induces oxidative stress. The influence of ANTX-a on the biological nervous system has been extensively investigated because ANTX-a is toxic to this system; nervous system diseases are characterized by disordered muscle bundles, decreased motor ability, tremor and gait changes, irregular breathing (Rymuszka and Sieroslawska, 2009). However, studies on the toxic effects of ANTX-a have focused on its neurotoxicity, but studies on ANTX-a-induced immunotoxicity have rarely been conducted. Moreover, little was known about its effects on the immune system of fish. Similar to other cyanotoxins, ANTX-a is extensively distributed in natural waters worldwide; this phenomenon has prompted researchers to investigate ANTX-a in natural waters. Ruiz et al. (2013) found ANTX-a for the first time in fresh water in South America. Heldman et al. (2008) reported the concentration of ANTX-a was 13 μg/L in a German lake, while the total concentration of ANTX-a in water reached 1750 μg/L in the United States (Bumke-Vogt et al., 1999). Considering that water quality is essential for fishes, researchers should investigate the immunotoxic effects of ANTX-a on fish.

Previous studies have shown that cyanotoxins, including nodularin and microcystins, can induce antioxidant system perturbation of *Carassius auratus*, resulting in oxidative stress (Sieroslawska and Rymuszka, 2019). Immune system perturbation caused by cyanotoxins can be indicated by the activity of antioxidant enzymes and levels of lipid peroxidation and reactive oxygen species (ROS) in immune cells (Huguet et al., 2019). As is well-known, overproduction of ROS or the decrease of cellular antioxidant levels can lead to the imbalance of cellular oxidation, which is called oxidative stress. Formation of ROS may lead to attack and destruction of important cell components, such as DNA and proteins, which may lead to permeability damage, membrane fluidity damage (Fernandez-Blanco et al., 2015). Production of ROS can also be stimulated by exposure to ANTX-a, thereby resulting in oxidative stress. Teneva et al. (2005) revealed that ANTX-a can induce the generation of ROS in cultured rat thymocytes. Antioxidant system perturbation is closely related to the occurrence of oxidative stress. As the main antioxidant enzymes in the first line of defense of the antioxidant system, catalase (CAT), superoxide dismutase (SOD), etc., can effectively scavenge excess oxygen free radicals in immune cells. Our previous study found that nodularin induced the apoptosis of *C. auratus* lymphocytes by oxidative stress and the mitochondrial apoptotic pathway (Zhang et al., 2012). In particular, it is necessary to study the response of the immune system of vertebrates to ANTX-a, especially in aquatic organisms.

This study utilized ANTX-a as the target pollutant of lymphocytes isolated from *C. auratus* for investigating the toxic effects of different exposure concentrations on immune cells *in vitro*. The results were discussed mainly from the aspect of antioxidant response to illuminate the mechanism of oxidative stress in lymphocytes.

## Materials and Methods

### Chemicals and the Toxin

Purified ANTX-a (CAS No. 64285-06-9, C_10_H_15_NO, MW = 165.23) was purchased from Sigma (St. Louis, MO, United States). Lymphoprep was purchased from Huadong Pharmaceutical (Zhejiang, China). Fetal calf serum (FCS) and RPMI-1640 medium were acquired from Hangzhou Key Shengwu (Hangzhou, China). Malonaldehyde (MDA), SOD, glutathione (GSH), CAT, glutathione-s-transferase (GST), glutathione reductase (GR), and glutathione peroxidase (GPx) assay kits were all purchased from Nanjing Jiancheng Bioengineering, Inc. (Jiangsu, China).

3-(4,5-Dimethylthiazol-2-yl)-2,5-diphenyl tetrazolium bromide, propidium iodide (PI), and rhodamine 123 assay kits were obtained from Beyotime Institute of Biotechnology (Shanghai, China). Other reagents used were purchased from commercial sources unless otherwise specified.

### Experimental Fish

From the hatchery of Freshwater Fisheries Research Institute (Zhejiang, China), we obtained about 400–500 g of bisexual *C. auratus* (6–10 months old). All experimental fish were raised and kept in circulating water with indoor temperature controlled at 25 ± 1°C. Feed fish with pellet feed at a daily ration of 0.7% of their body weight. After 2 weeks, healthy fish were utilized for subsequent studies.

### Lymphocyte Isolation and Cell Culture

The method of isolating lymphocytes was based on that of Zhang et al. (2008). *C. auratus* were sacrificed by decapitation, and their kidneys were removed. The mixed tissues were passed through the nylon screen. The cells were washed twice in serum-free cold medium and layered onto 1.5 volumes of Lymphoprep (density adjusted to 1.077 g/mL). After centrifugation at 640 × *g* for 30 min, non-adherent lymphocytes were carefully obtained by washing with PBS solution three times. Finally, the cells were cultured in an antibiotic-free RPMI-1640 medium containing 5% FCS. Use a hemocytometer, the number of cells was counted. The obtained cells were divided into five groups and treated with different concentrations of ANTX-a for 12 h.

### Electron Microscopy Observation

The lymphocytes were washed with PBS and immobilized overnight in 2.5% glutaraldehyde at 4°C. The cells were washed three times in PBS (0.1 M, pH 7.0) for 15 min each time, and then immobilized at osmium tetroxide (1%) for 1–2 h. Then, in Epon 812, the treatments were dehydrated and embedded by a gradient alcohol series and acetone. Finally, ultra-thin sections were prepared and stained with lead citrate and uranyl acetate, and then viewed under a transmission electron microscope (Philips, TECNAL-10).

### DNA Ladder Assay

A DNA ladder assay was performed via gel electrophoresis, as previously described. All groups of exposed lymphocytes were acquired and washed twice with cold PBS. Intracellular DNAs were then extracted utilizing an AxyPrep genomic DNA mini kit purchased from Axygen Biotechnology (Hangzhou, China), and electrophoresed using an agarose gel (1%). Finally, the extracted samples were stained with ethidium bromide (30 μg/L) and visualized utilizing a Kodak Gel Logic 200 (Molecular Imaging, NY, United States) using 1 kb as a size marker.

### Apoptosis Detection by Flow Cytometry

The cells were treated with different ANTX-a concentrations for 12 h, washed with cold PBS, and fixed in ethanol (70%) at 4°C for 1 day. The detailed detection method mainly refers to the method of Tavakkol-Afshari et al. (2008). The lymphocytes were washed with PBS twice and treated with PI staining buffer (50 μg/mL) and RNase (0.1 μg/mL) at 20°C for 30 min. The cells were filtered using a BD Falcon circular tube (No. 352235, Becton Dickinson, Franklin Lakes, NJ, United States) before analysis with a Guava easyCyte 8HT flow cytometer (Merck Millipore, Darmstadt, Germany).

### Intracellular ROS Assay

Lymphocytes were collected and homogenized in cold PBS after incubating with different ANTX-a concentrations for 12 h. The cells were exposed to dichloro-dihydrofluorescein diacetate. The final concentration was set at 10 μM and maintained at the room temperature for 20 min. The lymphocytes were passed through a cell mesh and homogenized before detection because they may adhere to the walls of the centrifuge tubes. The lymphocytes were analyzed by flow cytometer (Guava EasyCyte 8HT). The excitation wavelength and emission wavelength were 488 and 525 nm, respectively.

### Detection of GSH and MDA Contents

Lymphocytes were obtained and homogenized by centrifugation at 640 × *g* for 15 min at 4°C in cold PBS. The GSH and MDA contents were determined according to the manufacturer’s instructions for the use of the assay kits (Jiancheng, Nanjing, China).

### Measurement of Antioxidant Enzyme Activities

The activities SOD, CAT, GR, GPx, and GST were detected in this study. SOD activity was detected according to the instructions in the enzyme activity assay kit (WST-1). Lymphocytes were obtained and homogenized by centrifugation at 640 × *g* for 15 min at 4°C in cold PBS to obtain the supernatant. The mixture was incubated at 37°C for 20 min. Finally, using a multimode microplate reader (Infinite M1000), the absorbance was measured at 450 nm. The sample activity was expressed in unit/mg protein. The activities of other enzymes, namely, CAT, GR, GPx, and GST, were detected according to the instructions indicated in the corresponding assay kits.

### Statistical Analysis

All experimental data were recorded and calculated in Microsoft Excel^®^ software, which were presented as the mean ± standard deviation (SD) of five independent experiments performed in duplicate and triplicate. The samples were analyzed by one-way ANOVA, and *p* < 0.05 and *p* < 0.01 were considered statistically significant.

## Results

### Ultrastructural Observations of Lymphocytes

**FIGURE 1:**
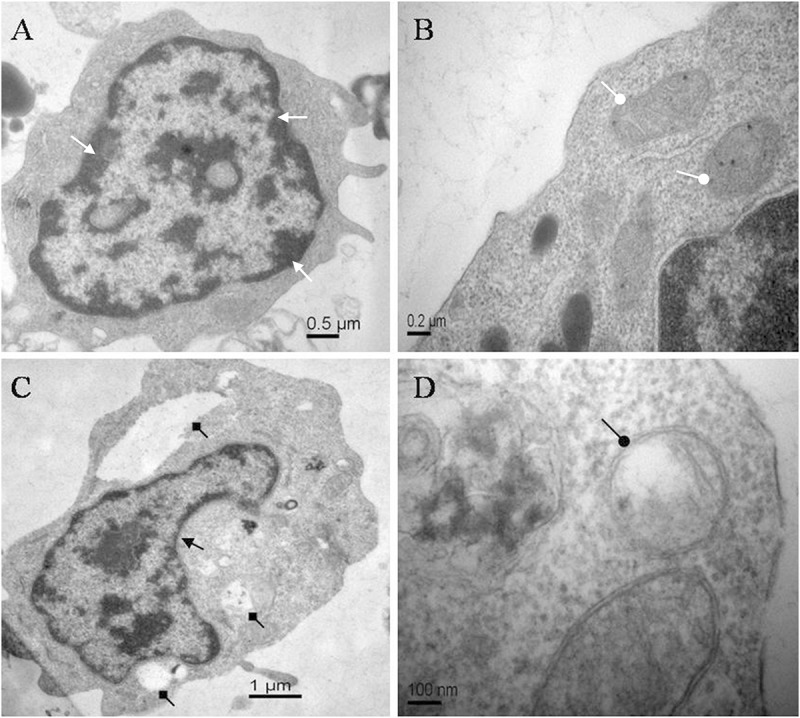
Electron micrographs of carp immunocytes treated with ANTX-a: **(A,B)** control cells showed homogeneous nuclear chromatin (white triangle arrow) and normal mitochondria (white circular arrow). **(C,D)** Cells treated with 100 μg/L ANTX-a for 12 h showed a condensed cytoplasm (black triangle arrow) with hollow bubbles (black square arrow) and slight swelling of the mitochondria (black circular arrow).

The morphological changes of lymphocytes of *C. auratus in vitro* treated with ANTX-a (0 and 100 μg/L) for 12 h were observed through transmission electron microscopy. Figures 1A,B show the normal lymphocytes of the control group, and Figures 1C,D show the lymphocytes exposed to 100 μg/L ANTX-a for 12 h. In the control group, the lymphocytes exhibited homogeneous nuclear chromatin (white triangle arrow, Figure 1A), and the mitochondria were not expanded (white circular arrow, Figure 1B). The morphological changes of lymphocytes exposed to 100 μg/L ANTX-a for 12 h were observed; condensed cytoplasm (black triangle arrow), slight swelling of the mitochondria (black circular arrow), and vacuolization (black square arrow) were observed in these lymphocytes (Figures 1C,D). These results indicated that ANTX-a damaged the lymphocytes of *C. auratus.*

### ANTX-a-Induced DNA Fragmentation in Lymphocytes

**FIGURE 2:**
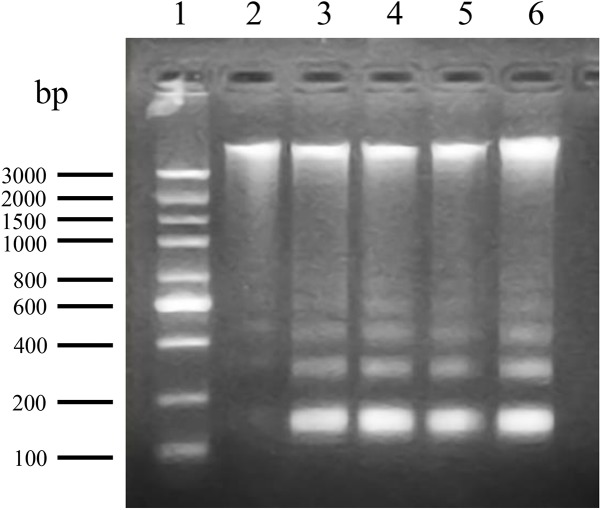
DNA fragmentation of carp immunocytes treated with ANTX-a determined by agarose gel electrophoresis. Lane 1: 100–3000 bp molecular size markers of DNA ladder; lane 2: DNA from control lymphocytes; lanes 3–6: DNA ladder patterns of immunocytes induced by ANTX-a for 12 h at concentrations of 0.01, 0.1, 1, and 10 mg/L, respectively.

Biochemically, the fragmentation of the nuclear chromatin of DNA is a typical character of apoptosis (Bortner et al., 1995), which leads to 180–200 bp fragments or complete DNA fragments. DNA fragmentation of ANTX-a-exposed cells was evaluated using agarose gel electrophoresis. The control only showed the total genome strip, and the total DNA extracted from cells exposed to different ANTX-a concentrations for 12 h distinctly showed a ladder pattern (Figure 2). These results demonstrated that ANTX-a can trigger apoptosis in the lymphocytes of *C. auratus in vitro*.

### Flow Cytometric Determination of ANTX-a-Induced Apoptosis

**FIGURE 3:**
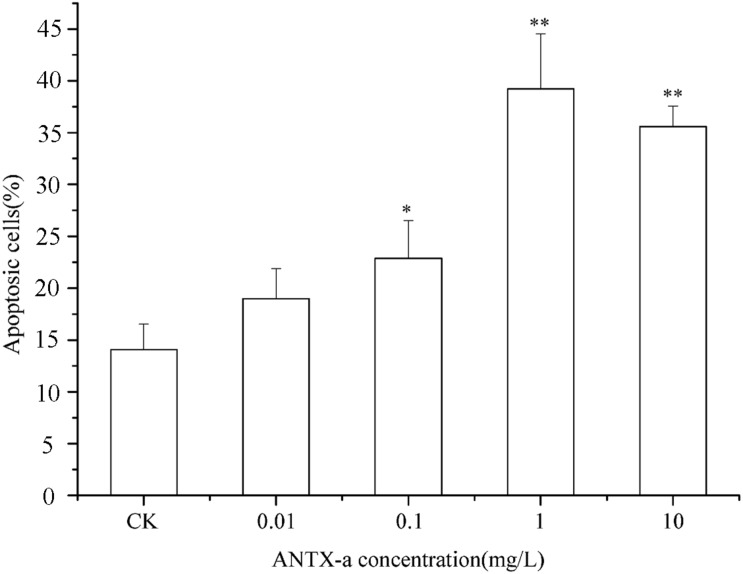
Apoptotic effects of carp immunocytes were detected after treatment with 0.01, 0.1, 1, and 10 mg/L ANTX-a for 12 h. Data represent the mean ± SD, and significant differences from the control were determined as *p < 0.05 and **p < 0.01.

The percentages of apoptotic cells exposed to various concentrations of ANTX-a *in vitro* are shown in Figure 3. After 12 h of exposure, the percentages initially increased as the ANTX-a concentration increased. At > 1 mg/L ANTX-a, the percentages of apoptotic cells decreased; the percentages in the treatment group with a high ANTX-a concentration (1 mg/L) increased 1.08-fold compared with that of the untreated group (*p* < 0.01). These results suggested that ANTX-a causes the apoptosis of fish lymphocytes. Necrosis occurred when the exposure concentration was 10 mg/L.

### Analysis of ROS and MDA Contents

**FIGURE 4:**
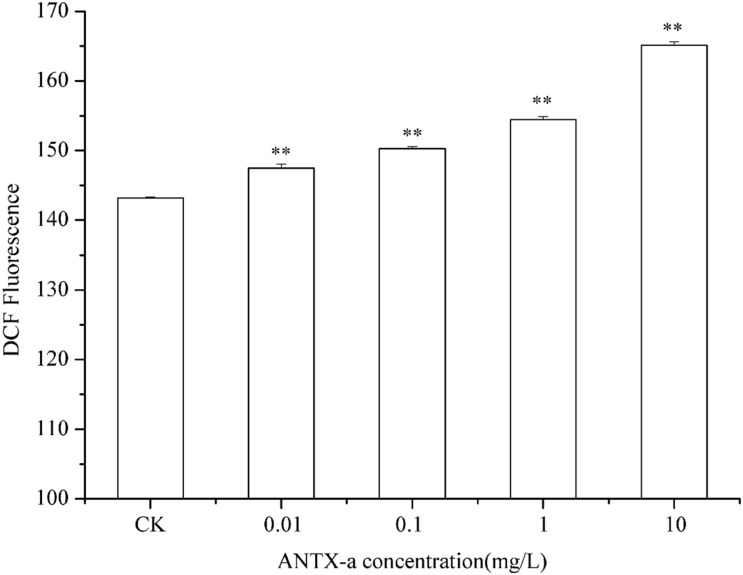
Changes in intracellular ROS levels of carp immunocytes were determined after cells were treated with ANTX-a (0.01, 0.1, 1, and 10 mg/L) for 12 h. Data represent the mean ± SD, and a significant difference from the control was determined as **p < 0.01.

**FIGURE 5:**
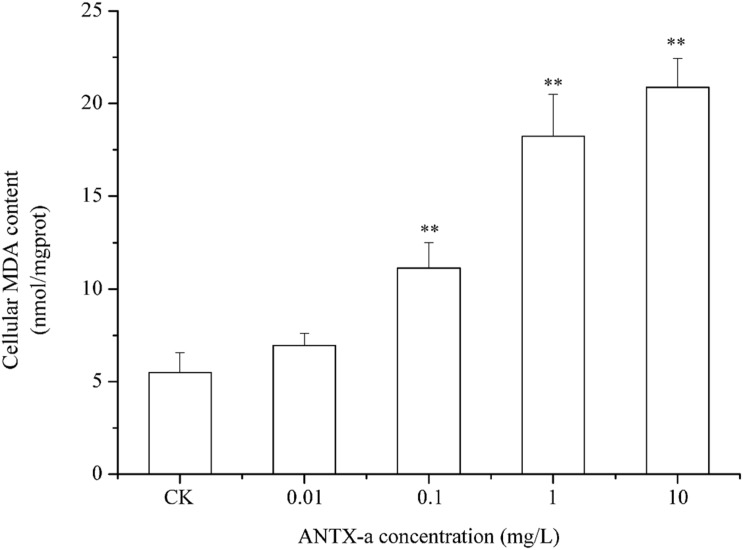
Dose-dependent effects of ANTX-a on MDA content in carp immunocytes. Cells were treated with 0.01, 0.1, 1, and 10 mg/L ANTX-a for 12 h. Data represent the mean ± SD, and a significant difference from the control was determined as **p < 0.01.

The ROS contents in fish lymphocytes treated with different concentrations of ANTX-a *in vitro* for 12 h were measured using flow cytometry. Figure 4 shows that the ROS levels increased with increasing ANTX-a concentration. The ROS content of the group exposed to the lowest concentration of ANTX-a (0.01 mg/L) significantly increased compared with that of the control (*p* < 0.01). The generation of intracellular ROS induced by ANTX-a exhibited a dose-dependent increase. According to Figure 5, ANTX-a induced MDA formation in the lymphocytes. The MDA level of the 0.1 mg/L ANTX-a-treated group was more than twofold higher than that of the control (*p* < 0.01). Overall, the elevation of MDA induced by ANTX-a was also dose-dependent.

### Determination of SOD and CAT Activities

**FIGURE 6:**
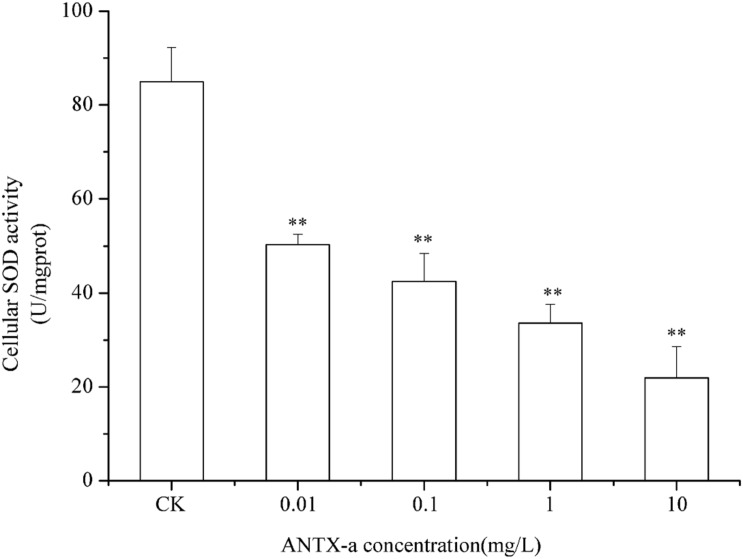
SOD activity in carp immunocytes exposed to ANTX-a for 12 h. ANTX-a concentrations were 0.01, 0.1, 1, and 10 mg/L. Dose-dependent effects were also detected. Data represent the mean ± SD, and a significant difference from the control was determined as **p < 0.01.

**FIGURE 7:**
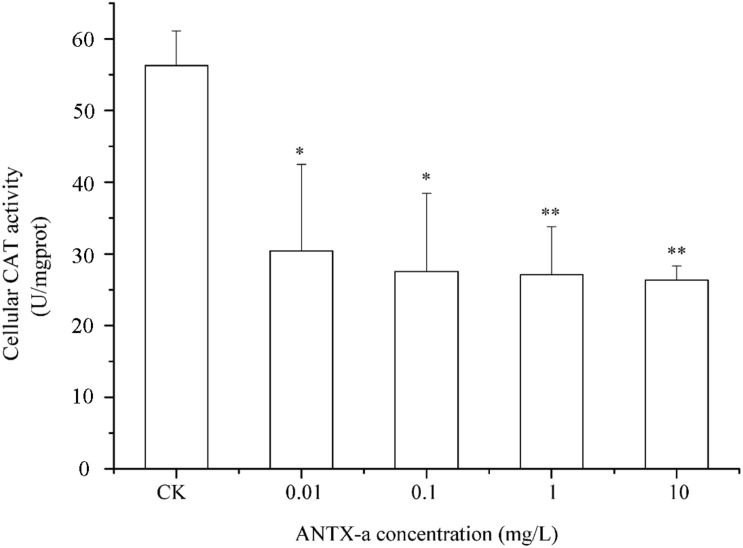
Changes in CAT activity after carp immunocytes were treated with 0.01, 0.1, 1, and 10 mg/L ANTX-a for 12 h. CAT activity was determined using a CAT activity kit. Data represent the mean ± SD, and a significant difference from the control was determined as *p < 0.05 and **p < 0.01.

SOD and CAT are critical antioxidant enzymes in cells. After the fish lymphocytes were exposed to different concentrations of ANTX-a for 12 h, the activities of SOD and CAT were, respectively, detected (Figures 6, 7). Figure 6 shows that SOD activity in fish lymphocytes significantly decreased with increasing ANTX-a exposure concentration. The activity of SOD of the 0.01 mg/L ANTX-a -treated group decreased by 41% compared with that of the untreated group. As shown in Figure 7, the CAT activity of lymphocytes in all of the ANTX-a-treated groups decreased. Compared with that of the untreated groups, the CAT activity decreased by 46% (*p* < 0.05) in the group exposed to 0.01 mg/L ANTX-a.

### GSH Levels in Fish Lymphocytes

**FIGURE 8:**
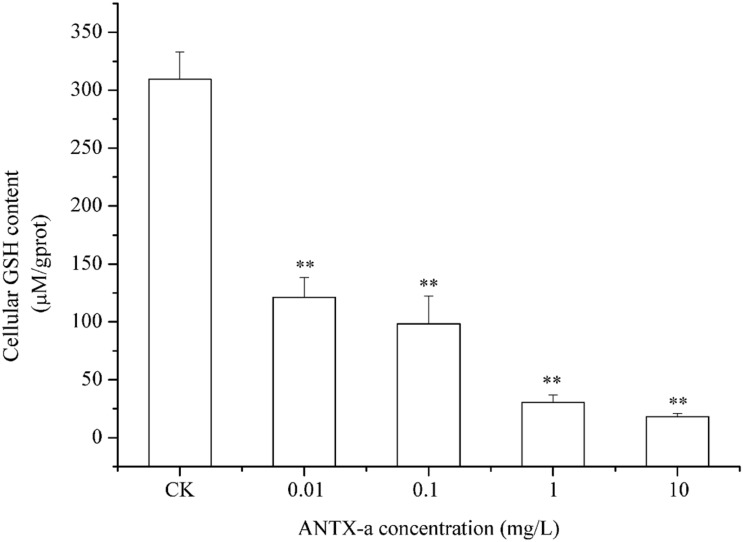
ANTX-a effects on GSH levels in carp immunocytes after treatment with ANTX-a for 12 h. GSH contents were detected to have dose-dependent effects. Data represent the mean ± SD, and a significant difference from the control was determined as **p < 0.01.

In the dose-dependent experiment, the GSH levels in each group are shown in Figure 8, in which the GSH content distinctly decreased in all ANTX-a-treated groups. The GSH content of the group treated with the lowest ANTX-a concentration (0.01 mg/L) decreased significantly by about 61% compared with that of the control group (*p* < 0.01). All of these results indicated that GSH levels in ANTX-a-treated lymphocytes were also dose-dependent.

### Analysis of GR, GPx, and GST Activities in Fish Lymphocytes

**FIGURE 9:**
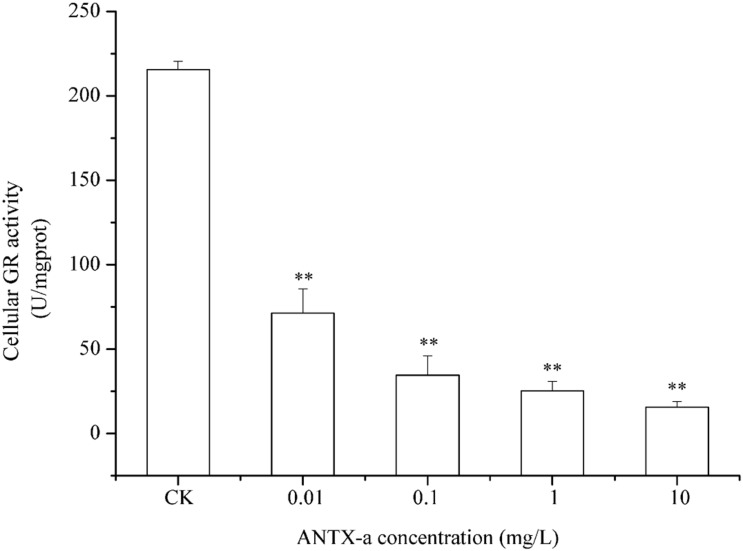
GR activity in carp immunocytes after exposure to ANTX-a for 12 h. The exposure concentrations of ANTX-a were 0.01, 0.1, 1, and 10 mg/L. Data represent the mean ± SD, and a significant difference from the control was determined as **p < 0.01.

**FIGURE 10:**
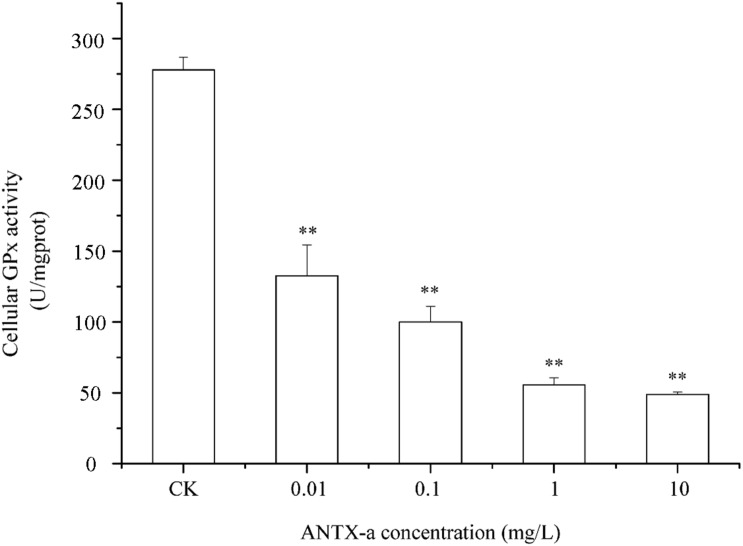
The intracellular GPx activity in carp immunocytes was determined after cells were treated with ANTX-a (0.01, 0.1, 1, and 10 mg/L) for 12 h. Data represent the mean ± SD, and a significant difference from the control was determined as **p < 0.01.

**FIGURE 11:**
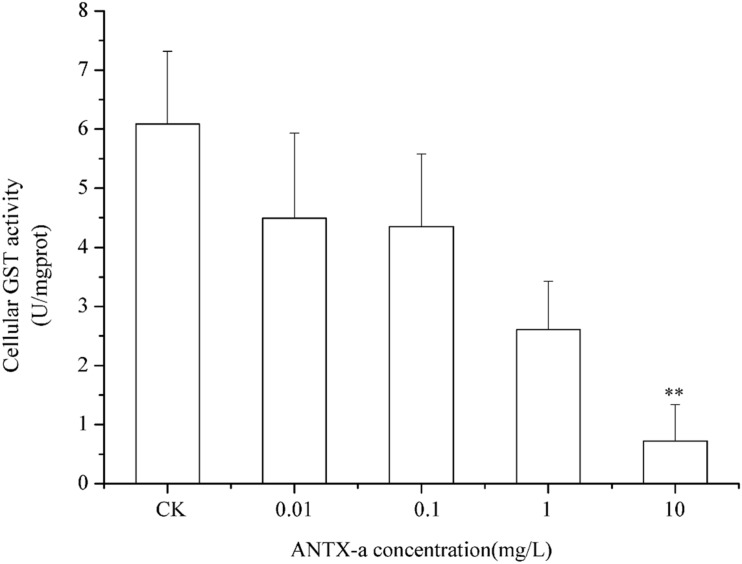
GST activity in cells treated with ANTX-a for 12 h. ANTX-a concentrations were 0.01, 0.1, 1, and 10 mg/L. Data represent the mean ± SD, and a significant difference from the control was determined as **p < 0.01.

Figures 9–11 show the activities of GR, GPx, and GST in fish lymphocytes exposed *in vitro* to varying ANTX-a concentrations for 12 h. The activities of GR and GPx in fish lymphocytes were sensitive to ANTX-a after 12 h of exposure regardless of treatment concentration. Figures 9, 10 show that the activities of GR and GPx significantly decreased as the ANTX-a concentration increased during 12 h of exposure; the activities of GR and GPx decreased by approximately 67 and 54% in the 0.01 mg/L treatment group compared with those of the control, respectively (*p* < 0.01). Figure 11 shows that GST activities decreased in all ANTX-a-treated groups; the GST activity in cells treated with 10 mg/L ANTX-a significantly decreased compared with that in the untreated group (*p* < 0.01). Thus, the activities of GR, GPx, and GST were observed to be affected by ANTX-a in a dose-dependent manner.

## Discussion

As a neurotoxin, the potential threat of ANTX-a to the immune system of aquatic organisms has long been underestimated. Lymphocytes in fish, which are very sensitive to water pollution, play a vital role in enhancing adaptive immunity, regulating tissue inflammation and host protection (Vivier et al., 2018). Most effects of ANTX-a exposure on the morphological and functional tissues of animals involve lymphocytes, such as low-dose ANTX-a-induced inflammation and apoptosis of immune cells and mouse brain cells (Takser et al., 2016). Although ANTX-a shows widespread occurrence and is highly toxic to animals, little is known about its mechanism of action and biotransformation in vertebrates, especially its toxic mechanisms underlying its impact on the fish immune system. Thus, we used the lymphocytes as our study object, and we detected the activities of major antioxidant enzymes to evaluate the toxic effects and related mechanisms of ANTX-a on fish.

In this study, the apoptosis of fish lymphocytes after ANTX-a exposure was investigated. In addition, our results showed that ANTX-a exposure activated major antioxidant enzymes (e.g., CAT, SOD) and induced the accumulation of MDA and ROS by damaging the activities of SOD, CAT, GSH, GR, and GPx. To illustrate the types of lymphocytes apoptosis, the changes in intracellular DNA and organelles were analyzed. As shown in Figures 1, 2, marked DNA fragmentation, condensed cytoplasm, and swollen mitochondria were observed in the highest concentration group (10 mg/L), which was similar to our previous research (Zhang et al., 2012). DNA fragmentation is a great description of biochemical changes in apoptosis (Wyllie, 1980). Thus, the apoptosis of fish lymphocytes which induced by ANTX-a may have been attributed to mitochondrial damage and DNA fragmentation. ANTX-a at 10 mg/L was reported to reduce the ATP level of leukocytes *in vitro*, and a concentration-dependent reduction in the proliferative capacity of T and B lymphocytes has also been observed (Bownik et al., 2012).

In order to expound the mechanism of mitochondrial damage induced by ANTX-a, ROS, and MDA, indices of oxidative stress were determined in our study. ROS, including superoxide anion radicals, hydrogen peroxide, and hydroxyl radicals, which are created by aerobic organisms, particularly in the mitochondrial respiratory chain, play a major role in hypoxia adaptation and cell proliferation (Zhao et al., 2019). However, excessive ROS may lead to irreversible cell damage and even cell death, which is called oxidative stress. Lipid peroxidation (measured as MDA), which is used as a biomarker for the degree of oxidative stress and damage, affecting membrane mobility and the integrity of membrane-related biomolecule (Wei and Yang, 2015). Many studies report that chemical environmental pollutants can induce ROS production in fish cells (Garcia-Gomez et al., 2020). Gagnaire et al. (2019) revealed that DNA damage and ROS production were caused by the exposure of tritiated water. Our previous studies also showed the increase of ROS in cells after exposed to nodularin and microcystins (Zhang et al., 2014a). Likewise, exposure to ANTX-a for 12 h in our study induced a dose-dependent increase in the level of ROS and MDA in fish lymphocytes compared with those of the untreated group (Figures 4, 5). Microcystin-LR can trigger intestinal DNA damage caused by excess ROS (Wen et al., 2019); saxitoxin can promote MDA production to induce lipid peroxidation (Melegari et al., 2012). It is apparent that high concentrations of ROS and MDA can be induced by cyanotoxins, and the generation of ROS is always accompanied by MDA products. These results indicated that ANTX-a leads to excessive ROS production resulting in oxidative stress.

However, occurrence of oxidative stress is closely related to the antioxidant system. CAT and SOD, which decompose hydrogen peroxide and superoxide anions, are important in the first defense line of organisms against excess free radicals (Yang et al., 2014). SOD activity correlates well with the immune competence of aquatic organisms because SOD is significant in minimizing the oxidative damage to host cells in immune defenses (Ali et al., 2020). Poniedzialek et al. (2015) suggested that the exposure of cylindrospermopsin (CYN) induces a dose-dependent increase in H_2_O_2_ concentration in 0.5 h, and when the concentration of H_2_O_2_ was highest, the activities of SOD and CAT decreased significantly. Similarly, in our study, Figures 6, 7 show that the activities of SOD and CAT in fish lymphocytes were all reduced in the lowest ANTX-a exposure group (0.01 mg/L) compared with those in the untreated groups, and the changes in SOD activity were more obvious than those in CAT activity. Pinho et al. (2005) discovered that at the end of the experiment, CAT activity was found to be significantly reduced in crabs exposed to the highest dose of *Microcystis aeruginosa* extract. Excessive ROS can inhibit the activity of CAT, and the inhibition of CAT activity directly leads to an increase in H_2_O_2_, which results in the inhibition of SOD activity, thus forming a vicious cycle (Hernandez et al., 2013). In addition, the enzyme activities of the endogenous antioxidant defense system reduced in the kidney of mice treated with Microcystin-leucine arginine (Jos et al., 2005). These results demonstrated that ATNX-a can reduce the activities of antioxidant enzymes, which is also the reason why oxidative stress occurred in fish lymphocytes.

Throughout the entire antioxidant protection process, GR can catalyze GSSG to form GSH, and GPX catalysis can help GSH to remove H_2_O_2_ from cells. GSH, an important non-enzyme antioxidant substance in cells, can prevent oxidative damage caused by reactive oxidants, can directly or indirectly maintain intracellular oxidative balance, and plays a significant role in protecting cells from oxidative stress (Ali et al., 2020). Rymuszka and Sieroslawska (2018) revealed that in primary leukocytes and small-cell lung cancer cells exposed to nodularin (0.01–0.1 mu g/mL), intracellular ROS production increased significantly and GSH levels decreased. As is shown in our results, the GSH content and the activities of its corresponding enzymes GPx and GR were significantly reduced in the lymphocytes compared to those in the untreated group (Figures 8–10); the activities of GR and GPx decreased by approximately 67 and 54% in the 0.01 mg/L treatment group compared with those in the control, respectively (*p* < 0.01). The decrease in GPx activity may be due to the reduced GSH content, as previously demonstrated by Li et al. (2003). As an antioxidant enzyme that catalyzes the conversion of NADPH-dependent oxidized GSH (GSSG) to GSH, the loss of GR activity is a direct reason for the reduced GSH levels. Therefore, a reduction in the conversion of GSSG back to its reduced form (GSH) occurred following the low levels of GSH. Our previous study indicated that nodularin (100 μg/L) induced oxidative damage of fish lymphocytes via a reduced GSH content and GPx activity (Zhang et al., 2014b).

In the present study, antioxidant and relevant enzymes were all reduced; compared with the that of the control, the GR and GPx activities of the 0.01 mg/L treatment group were reduced by approximately 67 and 54%, respectively (Figures 9, 10); thus, the antioxidant system of fish lymphocytes was destroyed by ANTX-a. In addition to GR and GPx, GST, as an enzyme mediator that binds GSH to hazardous compounds, plays a crucial role in the process of cell detoxification. As an electrophilic target, GSH can attack GSH nucleophiles by binding active sites, thus achieving antioxidant and detoxification functions (Mihaljevic et al., 2020). Figure 11 shows that GST activity in all fish lymphocytes exposed to ANTX-a *in vitro* for 12 h decreased in a dose-dependent manner regardless of treatment concentration. The inactivation of GST corresponded to changes in the antioxidant system. Cylindrospermopsin-administered fish showed a decrease in GST activity after 7 days of exposure (da Silva et al., 2018). However, Freitas et al. (2015) discovered that MC-LR and CYN can significantly increase GST activities in lettuce roots. Therefore, symbiosis with plants may indicate that aquatic organisms are more vulnerable to exposure to algal toxins. For the final oxidative stress parameter evaluated in our experiment, the changes in ROS contents were significantly less than those in antioxidant enzyme activities and antioxidants in fish lymphocytes; thus, ANTX-a induced apoptosis of fish immune cells mainly through disruption of the antioxidant system, which led to the overproduction of ROS and thus oxidative stress.

## Conclusion

This study demonstrated that neurotoxic ANTX-a exhibited immunotoxicity in carp lymphocytes by induction of apoptosis in an apparent dose-dependent manner. Intracellular ROS, MDA, and contents and reduced activity of antioxidant enzymes, such as SOD, GST, and CAT, are the main biomarkers of the cytotoxicity of ANTX-a to carp immunocytes *in vitro*, which were also measured to assess the influence of ANTX-a on fish lymphocytes and to investigate the potential mechanism of ANTX-a-induced immunotoxicity. These results suggested that ANTX-a induces the apoptosis of fish immune cells mainly by destroying the antioxidant system, causing excessive ROS production, and, finally, inducing oxidative stress. Further studies are needed to expound the exact mitochondrion apoptotic pathway involved in the apoptosis of ANTX-a induced in fish lymphocytes.

## Data Availability Statement

All datasets generated for this study are included in the article/supplementary material.

## Ethics Statement

The animal study was reviewed and approved by the Experimental Animal Ethics Committee of Hangzhou Normal University.

## Author Contributions

YZ designed the study, writing, and interpretation of the results. LS, XY, DZ, YH, YL, YD, WZ, and JD carried out most of the experimental work. HZ had overall responsibility for the project and editing the whole manuscript.

## Conflict of Interest

The authors declare that the research was conducted in the absence of any commercial or financial relationships that could be construed as a potential conflict of interest.

## References

[B1] Ali S. S. Ahsan H. Zia M. K. Siddiqui T. Khan F. H. (2020). Understanding oxidants and antioxidants: classical team with new players. *J. Food Biochem.* 44:e13145 10.1111/jfbc.13145 31960481

[B2] Bortner C. D. Oldenburg N. B. E. Cidlowski J. A. (1995). The role of DNA fragmentation in apoptosis. *Trends Cell Biol.* 5 21–26. 10.1016/s0962-8924(00)88932-1 14731429

[B3] Bownik A. Rymuszka A. Sieroslawska A. Skowronski T. (2012). Anatoxin-a induces apoptosis of leukocytes and decreases the proliferative ability of lymphocytes of common carp (Cyprinus carpio L.) in vitro. *Pol. J. Vet. Sci.* 15 531–535. 10.2478/v10181-012-0082-7 23214375

[B4] Bumke-Vogt C. Mailahn W. Chorus I. (1999). Anatoxin-a and neurotoxic Cyanobacteria in German lakes and reservoirs. *Environ. Toxicol.* 14 117–125. 10.1002/(SICI)1522-7278(199902)14:13.0.CO;2-V

[B5] Carneiro M. Gutierrez-Praena D. Osorio H. Vasconcelos V. Carvalho A. P. Campos A. (2015). Proteomic analysis of anatoxin-a acute toxicity in zebrafish reveals gender specific responses and additional mechanisms of cell stress. *Ecotoxicol. Environ. Saf.* 120 93–101. 10.1016/j.ecoenv.2015.05.031 26046835

[B6] Chia M. A. Kramer B. J. Jankowiak J. G. Bittencourt-Oliveira M. D. C. Gobler C. J. (2019). The individual and combined effects of the cyanotoxins, anatoxin-a and microcystin-LR, on the growth, toxin production, and nitrogen fixation of prokaryotic and eukaryotic algae. *Toxins* 11:43 10.3390/toxins11010043 PMC6357180 30650515

[B7] Cires S. Ballot A. (2016). A review of the phylogeny, ecology and toxin production of bloom-forming *Aphanizomenon* spp. and related species within the Nostocales (cyanobacteria). *Harmful Algae* 54 21–43. 10.1016/j.hal.2015.09.007 28073477

[B8] da Silva R. D. C. Grotzner S. R. Moura Costa D. D. Esquivel Garcia J. R. Muelbert J. (2018). Comparative bioaccumulation and effects of purified and cellular extract of cylindrospermopsin to freshwater fish *Hoplias malabaricus*. *J. Toxicol. Environ. Health Part A Curr. Issues* 81 620–632. 10.1080/15287394.2018.1469101 29764335

[B9] Du X. Liu H. Yuan L. Wang Y. Ma Y. Wang R. (2019). The diversity of cyanobacterial toxins on structural characterization, distribution and identification: a systematic review. *Toxins* 11:530 10.3390/toxins11090530 PMC6784007 31547379

[B10] Fernandez-Blanco C. Font G. Ruiz M.-J. (2015). Oxidative DNA damage and disturbance of antioxidant capacity by alternariol in Caco-2 cells. *Toxicol. Lett.* 235 61–66. 10.1016/j.toxlet.2015.03.013 25827405

[B11] Fitzgeorge R. B. Clark S. A. Keevil C. W. (1994). “Routes of intoxication,” in *Detection Methods for Cynobacterial Toxins*, eds Codd G. A. Jefferies T. M. Keevil C. W. (Cambridge: The Royal Society of Chemistry), 69–74.

[B12] Freitas M. Azevedo J. Pinto E. Neves J. Campos A. Vasconcelos V. (2015). Effects of microcystin-LR, cylindrospermopsin and a microcystin-LR/cylindrospermopsin mixture on growth, oxidative stress and mineral content in lettuce plants (*Lactuca sativa* L.). *Ecotoxicol. Environ. Saf.* 116 59–67. 10.1016/j.ecoenv.2015.02.002 25768423

[B13] Gagnaire B. Arcanjo C. Cavalie I. Camilleri V. Simon O. Floriani M. (2019). Tritiated water exposure in zebrafish, Danio rerio: effects on the early-life stages. *Environ. Toxicol. Chem.* 39 648–658. 10.1002/etc.4650 31858643

[B14] Garcia-Gomez C. Garcia S. Obrador A. Almendros P. Gonzalez D. Dolores Fernandez M. (2020). Effect of ageing of bare and coated nanoparticles of zinc oxide applied to soil on the Zn behaviour and toxicity to fish cells due to transfer from soil to water bodies. *Sci. Total Environ.* 706:135713 10.1016/j.scitotenv.2019.135713 31791765

[B15] Heldman C. J. Krick W. R. Perkins D. A. K. Harrahy E. A. Sonzogni W. C. (2008). New measurements of cyanobacterial toxins in natural waters using high performance liquid chromatography coupled to tandem mass spectrometry. *J. Environ. Q.* 37 1817–1824. 10.2134/jeq2007.0368 18689743

[B16] Hernandez A. F. Lacasana M. Gil F. Rodriguez-Barranco M. Pla A. Lopez-Guarnido O. (2013). Evaluation of pesticide-induced oxidative stress from a gene-environment interaction perspective. *Toxicology* 307 95–102. 10.1016/j.tox.2012.09.007 23032575

[B17] Huguet A. Lanceleur R. Quenault H. Le Hegarat L. Fessard V. (2019). Identification of key pathways involved in the toxic response of the cyanobacterial toxin cylindrospermopsin in human hepatic HepaRG cells. *Toxicol. Vitro.* 58 69–77. 10.1016/j.tiv.2019.03.023 30905859

[B18] Ibelings B. W. Havens K. E. (2008). “Cyanobacterial toxins: a qualitative meta-analysis of concentrations, dosage and effects in freshwater, estuarine and marine biota,” in *Cyanobacterial Harmful Algal Blooms: State of the Science and Research Needs*, ed. Hudnell H. K. (Berlin: Springer), 675–732. 10.1007/978-0-387-75865-7_32 18461789

[B19] Jos A. Pichardo S. Prieto A. I. Repetto G. Vazquez C. M. Moreno I. (2005). Toxic cyanobacterial cells containing microcystins induce oxidative stress in exposed tilapia fish (*Oreochromis* sp.) *under laboratory conditions*. *Aquat. Toxicol.* 72 261–271. 10.1016/j.aquatox.2005.01.003 15820106

[B20] Li X. Liu Y. Song L. Liu J. (2003). Responses of antioxidant systems in the hepatocytes of common carp (*Cyprinus carpio* L.) to the toxicity of microcystin-L R. *Toxicon* 42 85–89. 10.1016/s0041-0101(03)00104-1 12893065

[B21] Loftin K. A. Graham J. L. Hilborn E. D. Lehmann S. C. Meyer M. T. Dietze J. E. (2016). Cyanotoxins in inland lakes of the United States: occurrence and potential recreational health risks in the EPA National Lakes Assessment 2007. *Harmful Algae* 56 77–90. 10.1016/j.hal.2016.04.001 28073498

[B22] Melegari S. P. Perreault F. Moukha S. Popovic R. Creppy E. E. Matias W. G. (2012). Induction to oxidative stress by saxitoxin investigated through lipid peroxidation in Neuro 2A cells and *Chlamydomonas reinhardtii* alga. *Chemosphere* 89 38–43. 10.1016/j.chemosphere.2012.04.009 22546629

[B23] Metcalf J. S. Banack S. A. Powell J. T. Tymm F. J. M. Murch S. J. Brand L. E. (2018). Public health responses to toxic cyanobacterial blooms: perspectives from the 2016 Florida event. *Water Policy* 20 919–932. 10.2166/wp.2018.012

[B24] Mihaljevic I. Basica B. Marakovic N. Kovacevic R. Smital T. (2020). Interaction of organotin compounds with three major glutathione S-transferases in zebrafish. *Toxicol. Vitro* 62:104713 10.1016/j.tiv.2019.104713 31706034

[B25] Pinho G. L. L. da Rosa C. M. Maciel F. E. Bianchini A. Yunes J. S. Proenca L. A. O. (2005). Antioxidant responses and oxidative stress after microcystin exposure in the hepatopancreas of an estuarine crab species. *Ecotoxicol. Environ. Saf.* 61 353–360. 10.1016/j.ecoenv.2004.11.014 15922801

[B26] Poniedzialek B. Rzymski P. Karczewski J. (2015). The role of the enzymatic antioxidant system in cylindrospermopsin-induced toxicity in human lymphocytes. *Toxicol. Vitro* 29 926–932. 10.1016/j.tiv.2015.03.023 25863213

[B27] Rodriguez I. Fraga M. Alfonso A. Guillebault D. Medlin L. Baudart J. (2017). Monitoring of freshwater toxins in European environmental waters by using novel multi-detection methods. *Environ. Toxicol. Chem.* 36 645–654. 10.1002/etc.3577 27505279

[B28] Ruibal-Conti A. L. Ruiz M. A. Rodriguez M. I. Lerda D. Romero M. D. (2019). Assessment of specific antibodies as biological indicators of human chronic exposure to microcystins. *Ecotoxicol. Environ. Saf.* 175 236–242. 10.1016/j.ecoenv.2019.03.071 30903879

[B29] Ruiz M. Galanti L. Ruibal A. L. Rodriguez M. I. Wunderlin D. A. Ame M. V. (2013). First report of microcystins and anatoxin-a co-occurrence in san roque reservoir (Cordoba, Argentina). *Water Air Soil Pollut.* 224:17 10.1007/s11270-013-1593-2

[B30] Rymuszka A. Sieroslawska A. (2009). The immunotoxic and nephrotoxic influence of cyanotoxins to vertebrates. *Central Eur. J. Immunol.* 34 129–136.

[B31] Rymuszka A. Sieroslawska A. (2018). Comparative studies on the cytotoxic effects induced by nodularin in primary carp leukocytes and the cells of the fish CLC line. *Toxicon* 148 7–15. 10.1016/j.toxicon.2018.04.001 29621526

[B32] Sieroslawska A. Rymuszka A. (2019). Assessment of the cytotoxic impact of cyanotoxin beta-N-methylamino-L-alanine on a fish immune cell line. *Aquat. Toxicol.* 212 214–221. 10.1016/j.aquatox.2019.05.012 31132739

[B33] Smith R. A. Lewis D. (1987). A rapid analysis of water for anatoxin a, the unstable toxic alkaloid from Anabaena flos-aquae, the stable non-toxic alkaloids left after bioreduction and a related amine which may be nature’s precursor to anatoxin a. *Vet. Hum. Toxicol.* 29 153–154. 10.1016/S0955-2863(00)00070-X 3107204

[B34] Svircev Z. Lalic D. Savic G. B. Tokodi N. Backovic D. D. Chen L. (2019). Global geographical and historical overview of cyanotoxin distribution and cyanobacterial poisonings. *Archiv. Toxicol.* 93 2429–2481. 10.1007/s00204-019-02524-4 31350576

[B35] Takser L. Benachour N. Husk B. Cabana H. Gris D. (2016). Cyanotoxins at low doses induce apoptosis and inflammatory effects in murine brain cells: Potential implications for neurodegenerative diseases. *Toxicol. Rep.* 3 180–189. 10.1016/j.toxrep.2015.12.008 28959538 PMC5615428

[B36] Tavakkol-Afshari J. Brook A. Mousavi S. H. (2008). Study of cytotoxic and apoptogenic properties of saffron extract in human cancer cell lines. *Food Chem. Toxicol.* 46 3443–3447. 10.1016/j.fct.2008.08.018 18790714

[B37] Teneva I. Mladenov R. Popov N. Dzhambazov B. (2005). Cytotoxicity and apoptotic effects of microcystin-LR and anatoxin-a in mouse lymphocytes. *Folia Biol.* 51 62–67. 10.14712/fb2005051030062 16045237

[B38] Trainer V. L. Hardy F. J. (2015). Integrative monitoring of marine and freshwater harmful algae in washington state for public health protection. *Toxins* 7 1206–1234. 10.3390/toxins7041206 25860160 PMC4417964

[B39] Vivier E. Artis D. Colonna M. Diefenbach A. Di Santo J. P. Eberl G. (2018). Innate lymphoid cells: 10 years on. *Cell* 174 1054–1066. 10.1016/j.cell.2018.07.017 30142344

[B40] Wei K. Yang J. (2015). Oxidative damage of hepatopancreas induced by pollution depresses humoral immunity response in the freshwater crayfish *Procambarus clarkii*. *Fish Shellfish Immunol.* 43 510–519. 10.1016/j.fsi.2015.01.013 25655324

[B41] Wen C. Zheng S. Yang Y. Li X. Chen J. Wang X. (2019). Effects of microcystins-LR on genotoxic responses in human intestinal epithelial cells (NCM460). *J. Toxicol. Environ. Health Part A Curr. Issues* 82 1113–1119. 10.1080/15287394.2019.1698498 31818208

[B42] Wyllie A. H. (1980). Glucocorticoid-induced thymocyte apoptosis is associated with endogenous endonuclease activation. *Nature* 284 555–556. 10.1038/284555a0 6245367

[B43] Yang Y. Ma H. Zhou J. Liu J. Liu W. (2014). Joint toxicity of permethrin and cypermethrin at sublethal concentrations to the embryo-larval zebrafish. *Chemosphere* 96 146–154. 10.1016/j.chemosphere.2013.10.014 24184047

[B44] Zhang H. Fang W. Xiao W. Lu L. Jia X. (2014a). Protective role of oligomeric proanthocyanidin complex against hazardous nodularin-induced oxidative toxicity in *Carassius auratus* lymphocytes. *J. Hazard. Mater.* 274 247–257. 10.1016/j.jhazmat.2014.04.020 24794815

[B45] Zhang H. Wu Y. Fang W. Wang D. (2014b). Regulatory effect of quercetin on hazardous microcystin-LR-induced apoptosis of *Carassius auratus* lymphocytes in vitro. *Fish Shellfish Immunol.* 37 278–285. 10.1016/j.fsi.2014.02.015 24594009

[B46] Zhang H. Shao D. Wu Y. Cai C. Hu C. Shou X. (2012). Apoptotic responses of *Carassius auratus* lymphocytes to nodularin exposure in vitro. *Fish Shellfish Immunol.* 33 1229–1237. 10.1016/j.fsi.2012.08.016 22951228

[B47] Zhang H. Zhang J. Zhu Y. (2008). In vitro investigations for the QSAR mechanism of lymphocytes apoptosis induced by substituted aromatic toxicants. *Fish Shellfish Immunol.* 25:717 10.1016/j.fsi.2008.02.008 19004643

[B48] Zhao R.-Z. Jiang S. Zhang L. Yu Z.-B. (2019). Mitochondrial electron transport chain, ROS generation and uncoupling (Review). *Intern. J. Mol. Med.* 44 3–15. 10.3892/ijmm.2019.4188 PMC6559295 31115493

